# Integrating team resource management program into staff training improves staff’s perception and patient safety in organ procurement and transplantation: the experience in a university-affiliated medical center in Taiwan

**DOI:** 10.1186/1471-2482-14-51

**Published:** 2014-08-11

**Authors:** Ya-Chi Hsu, Jih-Shuin Jerng, Ching-Wen Chang, Li-Chin Chen, Ming-Yuan Hsieh, Szu-Fen Huang, Yueh-Ping Liu, Kuan-Yu Hung

**Affiliations:** 1 Center for Quality Management, National Taiwan University, Taipei, Taiwan; 2 Department of Internal Medicine, National Taiwan University Hospital and National Taiwan University College of Medicine, Taipei, Taiwan; 3 Department of Emergency Medicine, National Taiwan University Hospital and National Taiwan University College of Medicine, Taipei, Taiwan; 4 Department of Nursing, National Taiwan University Hospital and National Taiwan University College of Medicine, Taipei, Taiwan; 5 Department of Internal Medicine, National Taiwan University Hospital, No. 7, Zhongshan South Road, Taipei 100, Taiwan

**Keywords:** Organ transplantation, Teamwork, Team resource management, Patient safety

## Abstract

**Background:**

The process involved in organ procurement and transplantation is very complex that requires multidisciplinary coordination and teamwork. To prevent error during the processes, teamwork education and training might play an important role. We wished to evaluate the efficacy of implementing a Team Resource Management (TRM) program on patient safety and the behaviors of the team members involving in the process.

**Methods:**

We implemented a TRM training program for the organ procurement and transplantation team members of the National Taiwan University Hospital (NTUH), a teaching medical center in Taiwan. This 15-month intervention included TRM education and training courses for the healthcare workers, focused group skill training for the procurement and transplantation team members, video demonstration and training, and case reviews with feedbacks. Teamwork culture was evaluated and all procurement and transplantation cases were reviewed to evaluate the application of TRM skills during the actual processes.

**Results:**

During the intervention period, a total of 34 staff members participated the program, and 67 cases of transplantations were performed. Teamwork framework concept was the most prominent dimension that showed improvement from the participants for training. The team members showed a variety of teamwork behaviors during the process of procurement and transplantation during the intervention period. Of note, there were two potential donors with a positive HIV result, for which the procurement processed was timely and successfully terminated by the team. None of the recipients was transplanted with an infected organ. No error in communication or patient identification was noted during review of the case records.

**Conclusion:**

Implementation of a Team Resource Management program improves the teamwork culture as well as patient safety in organ procurement and transplantation.

## Background

Patient safety has become an important healthcare issue, as errors and adverse events remain difficult to completely eliminate that a number of measures have been advocated to reduce their occurrence [1-6]. Recognized by healthcare workers, including physicians [7], errors had been reportedly frequent experienced, and the majority of staff regarded 79% of these events as preventable, with a substantial portion of them considered as related to human factors [7-9]. Interventions to reduce the impact of human failures in the healthcare practice had been vigorously studied [8-10], including Team Resources Management (TRM) and TeamSTEPPS [2,5,9,10]. These programs and tools derived from aviation safety and crew training experiences [11,12], and focused on a number of training components for knowledge, skills and attitude, such as leadership, situation awareness, mutual support and communication [9,13], and have been deemed promising in enhancing patient safety in the healthcare settings.

Organ procurement and transplantation is a complex clinical process that involves interactions and collaboration among the members of multi-disciplinary teams in the healthcare system, typically across more than one clinical setting, and sometimes even more than one country. Team members typically are required to assure the completeness and accuracy while performing the task activities of the processes, with accurate collection and communication of patient information and data, but the nature of donation anonymous to the recipients imposes a limitation to sharing of information between the procurement and transplantation teams, which carries the risk for communication and information errors, which might result in subsequent harm to the patients, as exemplified by the reports and comments in the literature [14] in the cases of unintended transplantation of organs from HIV-positive donors [15,16], including a haunting case in Taiwan involved transmission of HIV to one heart, one liver, one lung and two kidney recipients [16]. The donor had a positive HIV test result, but during telephone communication for the report a key member of the procurement team mistakenly recorded the test result as “nonreactive” into the donor data system [16].

In an intervention aiming to improve the patient safety of organ procurement and transplantation, we integrated a TRM program into the training of the team members involved in the processes. During the intervention period, the team members were successfully provided with useful skills in teamwork, and were trained to apply the learned skills to their practice. Here we report our experience of this integration of the TRM program.

## Methods

### Setting

The intervention described below was an institution-initiated improvement program that management of the patients by the healthcare workers of the institution were mandatorily required to conform to the current and revised policies and procedures; therefore the Research Ethics Committee of the National Taiwan University Hospital waived the need for written informed consents from the patients and participating employee. By the time the study was carried out, the National Taiwan University Hospital (NTUH) was a 2,500 bed-, university-affiliated tertiary medical center in northern Taiwan. As a public, not-for profit healthcare organization, the hospital had 6,400 employees, including more than 1,000 physicians. All of the healthcare workers for the in-patient care, including the organ procurement and transplantation, were salaried and full-time. While transplantation had been a common service in NTUH for decades, potential donors were recruited both from the in-patients of this hospital as well as from other institutions through the Taiwan Organ Registry and Sharing Center (TORSC), which was established by the Department of Health of Taiwan Government in 2003. The Organ Procurement team then approached candidate donors through a standardized process, once aware of the information. After the validation of the feasibility of donation, including the confirmation of laboratory data to assure the eligibility for donation, the pertinent information was uploaded to the database of TORSC, through which a prioritized matched recipient was identified, and the transplantation was subsequently performed. The organ procurement team of NTUH approached about 70 potential donors each year, while the transplantation teams performed about 40 cases of organ transplantation each year.

### Intervention

This integrated program was based on the antecedent Team Resource Management (TRM) training courses in the institution, which was introduced in 2006 under collaboration with the lecturers from the China Airline, which provided Crew Resource Management program to enhance aviation safety in their organization. The TRM training program was originally designed to consist of a two-day seminar including a scenario-learning workshop, typically instructed by two senior physicians qualified by the Taiwan Joint Commission. Participants for the program included newly recruited resident doctors and interns, and members of the in-hospital resuscitation team, as a part of their on-job training. Before this study was performed, no teamwork train was required for the organ procurement and transplantation teams.

This 15-month intervention, which was carried out between November 2011 and January 2013 to improve the teamwork and patient safety for organ procurement and transplantation, included a series of education and practicing training courses consisting of four main components as shown in Table 1. Staff members were recruited from different disciplines who were involved in the processes of organ procurement and transplantation, including surgeons, operating room nurses, anesthesiologists, and laboratory technicians. The participants were arranged into three groups: the operating room and transplantation group, the organ procurement group and the laboratory group.

**Table 1:** Interventions on teamwork in organ procurement and transplantation

**Team resource management training course**	**Video demonstration and training**
• Lecture domains: leadership, communication, situation awareness, mutual support	• Video production of organ procurement and transplantation case presentation
• Sub-group discussion and concept formation of the group members	• Video demonstration and teaching of common TRM skills
• Case presentations by sub-groups	
**Skill development**	**Case review and feedback**
• Focused group interviews	• Periodic case reviews and meeting documentation
• TRM common skills demonstration by instructors	• Document review and feedback for assessment of TRM skill integration
• In-class evaluation of participants’ TRM skills by instructors	• Outcome measurement: skills usage and incidents related to teamwork

The first component of the TRM program, which was carried out during the first 6 months, included a combination of lectures and case-based interactive discussions in a simulative learning workshop, also summarized in Table 1. The lecture started with a special focus on safety issues in aviation, while the participants were then encouraged to reflectively think and discuss openly about the comparison between aviation service and hospital practice, stressing on possible errors or near misses during the processes. Participants were then randomly assigned to simulative learning workshops, where they practiced error and risk management skills, and shared personal experience on identification and management of near misses or errors. Essential concepts and knowledge related to the four domains of the TRM concepts [9], typically including leadership, communication, situation awareness and mutual support, were introduced during this period.

During the second component of the program, which spanned during the project months 7 to 9, focus group interviews were performed, and then the participants were provided with the main TRM training with the pertinent knowledge and commonly used skills. Stress was also put on the four dimensions of TRM, and skills such as briefing, debriefing, check-back, call-out and recognition of good communication as important to achieve the team goal, were also taught, as summarized in Table 1. Upon completion of workshop, the participants were expected to understand the benefits of incorporating the TRM skills into the real-life process during organ procurement and transplantation. At the end of the lectures, the instructors gave a debriefing summary and feedback to all participants who were then assigned into sub-teams with the goals to develop TRM-based checklists, working sheets, and re-designed organ procurement and transplantation processes. The staff members from the Center of Quality Management (including the authors of this study) observe the whole learning process and the behavioral content of the participants, while facilitating the teaching process in an encouraging fashion.

The third component was a video skill demonstration and training, which was carried out during the project months 10 to 12. Selected members of the participants made a video as a simulation of practice of the procurement and transplantation team. This video consisted of two parts: the former showed interactions among the team members with poor teamwork culture and unsafe practices, while the latter part became better in terms of teamwork and safety, demonstrating a number of pertinent skills related to the four dimension of TRM applied in the procurement and transplantation process. All of the participating workers then watched the video and discussed about the contents as an intended boost of teamwork culture after the previous teaching program was completed.

The fourth component consisted of case reviews and feedback activities, which spanned during the project months 4 to 15. After the completion of initial TRM training course, there remained a case discussion performed every 2 weeks in the first three months, which was carried out in addition to routine service process for the procurement and transplantation. All of the three groups of participants joined the discussion, and any near miss or error during the procurement or transplantation processes, in case identified, would be confirmed and documented at the meeting. The staff would then discuss the causes and possible solutions, and feedbacks would be sent to all of the team members. Reporting of the incidents was in a non-punitive fashion and could be anonymous in the cases concerned.

### Evaluation and analyses

To evaluate the effectiveness of the TRM program, we performed questionnaire interviews and document audits to assess the cognition and behavioral change of the participants. Monitoring for any near miss or error reported during the process was also performed on a real-time basis, which was handled mainly by members from the Center for Quality Management of the hospital.

The standardized questionnaire, consisting of two parts, was applied to evaluate the patient safety culture and learning perception about TRM for all participants. The first part consisted of 48 questions related to teamwork in five major categories, including teamwork framework, leadership, situation monitoring, communication between team members, and mutual support. The standardized questionnaire (designed in Chinese) used in this study was adopted from a project from the Department of Health of Taiwan (http://grbsearch.stpi.narl.org.tw/GRB_Search/grb/show_doc.jsp?id=1972573&q=*%3A*), of which tests for reliability and validity for the questionnaire were performed [see Additional file 1 for the detailed content originally in Chinese, and Additional file 2 as English translation]. In terms of reliability, the overall Cronbach’s alpha value was 0.955, with its value of 0.948 in teamwork framework, 0.921 in leadership, 0.937 in situation monitoring, 0.875 in communication, and 0.866 in team member interactions. For the overall validity, the chi square value from the indicated source questionnaire was 11193.40 (p < 0.001), with a CFI (comparative fit index) of 0.97, a CN (critical N) of 150.98, a GFI (goodness of fitting index) of 0.7, an AGFI (adjusted goodness of fitting index) of 0.67, an RMR (root mean square residual) of 0.092, an SRMR (standardized root mean square residual) of 0.055, and an RMSEA (root mean square error of approximation) of 0.094, suggesting an acceptable goodness of fitting. Answers from the participants were given by using a 7-point Likert-type scale (strongly disagree to strongly agree) sheet. The second part of the questionnaire was related to information pertinent to the participants including type of discipline, subspecialty, demographic characteristics, etc. The questionnaire interview was performed for every participant immediately before their attending the program and by the end of intervention.

Satisfaction of the participants with the TRM training program was also evaluated, using a satisfaction questionnaire with 5-point Likert-type scale (strongly disagree to strongly agree).

The investigators also performed a document review for the discussion and meeting records of the procurement and transplantation teams during the periodic meetings. The investigators retrieved pertinent information and discussed to identify and confirm about the findings of any skills related to TRM that were deemed apparently applied during the process of procurement and transplantation. The skills used and demonstrated in the records were then attributed to one of the four dimensions of TRM as provided during the training.

In addition, all reported near misses and errors to the Center for Quality Management of the institution were reviewed adequately to determine whether to relate the events to teamwork problem in the procurement and transplantation workers.

Results are presented as overall scores, average scores, and percentages for all questions. A paired *t*-test was used to compare the ratings before and after the TRM training course. Descriptive analysis of the demographics was reported as mean with standard deviation (SD). The Mann–Whitney U test was used for non-parametric analyses. Statistical analyses were performed using the SPSS 15.0 software (SPSS corp., Chicago, IL, USA), with p < 0.05 considered statistically significant.

## Results

The intervention was performed between November 2011 and January 2013. A total of 34 staff members participated this TRM program, including 23 (67.7%) from the operating room and transplantation group, 6 (17.7%) from the organ procurement group, and 5 (14.7%) from the laboratory group. Their mean duration of professional experience was 17.4 years, with 88.3% of them having worked for more than 3 years in this hospital, as shown in Table 2.

**Table 2:** Characteristics of the participants (n = 34) for TRM training course

**Characteristic**	**Number (%)**
Discipline	
Surgeon	12 (35.3)
Anesthesiologist	3 (8.8)
Nurses	15 (44.0)
Laboratory staff	4 (11.8)
Working unit	
Operating room	23 (67.7)
Organ procurement	6 (17.7)
Laboratory	5 (14.7)
Professional experience	
3 years or less	4 (11.8)
4 - 10 years	6 (17.7)
11 - 19 years	10 (29.4)
20 years or more	14 (41.2)

During the intervention period, there were 74 candidate donors who were managed with the organ procurement procedure, and a total of 117 sessions of laboratory tests were performed in the laboratories of this hospital for the blood samples sent to the laboratories from the candidate donors. Subsequently, there were 51 valid patients who actually successfully donated organs, and there were 88 recipients who underwent the organ transplantation procedure successfully in this hospital. All of the staff that had participated in the TRM training course did participate in the actual procurement or transplantation process, while the rest of staff members of the teams who did not participate in the training also performed routine practices during the process.

During the intervention period, there were 14 formal meetings, which included all relevant participants in this program, including team members from the OR and transplantation, procurement and laboratories, managers from the Center for Quality Management of this hospital. Staff members from the Information Technology Department also joined the meetings to provide necessary technical assistance. A platform for uploading relevant patient and clinical information was then established, while the team members were subsequently trained to use this platform to update information and to communicate with other members as a part of team interaction. The progresses were also reported to the Committee for Organ Transplantation Management in this hospital on a monthly basis for a total of 12 sessions, while the committee members provided oversight of the activities and implementation of improvement strategies. Monitored and audit data were also reported to the Committee as required.

### Evaluation of the training program

Survey of the satisfaction of the 34 participants to the TRM training program showed an overall satisfaction score of 3.97 ± 0.69, without significant difference among the three groups of staff (4.00 ± 0.20 in laboratory group, 3.96 ± 0.74 in OR group, and 3.95 ± 0.25 in procurement group, respectively).

### Effect of TRM training on teamwork attitude

**Figure 1:**
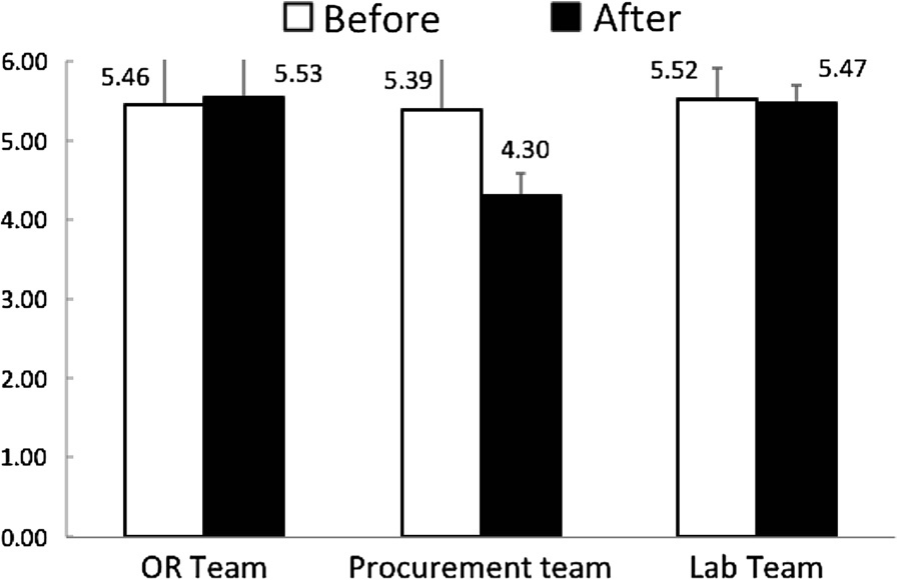
Changes of overall scores on perception of teamwork culture in the three groups of team members participating the TRM program (p > 0.05 in comparing changes in all three groups).

All of the 34 participants completed the before- and after-intervention questionnaire surveys, with valid data for analyses. The results are summarized in Table 3, which shows the highest score of perception was communication (5.83 ± 0.83), followed by teamwork framework (5.69 ± 0.90), while the lowest score was in mutual support (5.05 ± 0.96). Differences of the perception across the groups were not significant, either before or after the intervention. Effect of TRM training on teamwork perception for different teams was shown in Figure 1, showing a lack of significant change in the perception measurements, although that there was a trend of improvement in perception in the OR team, and a trend of worse perception in the procurement team. There was no significant change between the before- and after-intervention scores for overall as well as each one of the five dimensions, although the scores in teamwork framework, situation monitoring and mutual support appeared to be increased, while the scores in leadership and communication appeared to be decreased (p > 0.05 in all dimensions). The changes of the perceptions from the different teams in the five dimensions were no significant, despite Table 4 showed that the OR had a trend to improved teamwork framework perception (from 5.61 ± 0.87 to 5.86 ± 0.95) and situation monitoring (from 5.21 ± 0.95 to 5.41 ± 0.90), but the procurement team exhibited worse perceptions in all five dimensions after the intervention program.

**Table 3:** Comparison of teamwork perception before and after the TRM intervention

**Category**		**Rating (mean ± SD)**		***p***
		***Before (n = 34)***	***After (n = 34)***	
I	Teamwork framework	5.69 ± 0.90	5.82 ± 0.85	0.56
II	Leadership	5.52 ± 1.11	5.21 ± 1.11	0.26
III	Situation awareness	5.19 ± 1.01	5.25 ± 0.95	0.79
IV	Communication	5.83 ± 0.83	5.65 ± 0.96	0.41
V	Mutual support	5.05 ± 0.96	5.16 ± 1.18	0.68

**Table 4:** Changes of the perception scores after the intervention across the dimensions in different team groups

**Dimension/Team**	**Before**	**After**
Teamwork Framework		
OPR Team	5.61 ± 0.87	5.86 ± 0.95
Procurement Team	5.62 ± 1.21	5.18 ± 0.24
Laboratory Team	6.14 ± 0.59	5.88 ± 0.45
Leadership		
OPR Team	5.47 ± 1.12	5.31 ± 1.18
Procurement Team	5.40 ± 1.34	4.06 ± 1.06
Laboratory Team	5.90 ± 0.85	5.35 ± 0.28
Situation Monitoring		
OPR Team	5.21 ± 0.95	5.41 ± 0.90
Procurement Team	5.06 ± 1.36	3.73 ± 0.79
Laboratory Team	5.24 ± 1.05	5.38 ± 0.47
Communication		
OPR Team	5.83 ± 0.88	5.80 ± 0.90
Procurement Team	5.83 ± 0.83	4.00 ± 0.44
Laboratory Team	5.82 ± 0.74	4.97 ± 0.53
Mutual Support		
OPR Team	5.18 ± 0.99	5.24 ± 1.45
Procurement Team	5.03 ± 0.88	4.56 ± 0.69
Laboratory Team	4.50 ± 0.84	4.97 ± 0.79

The impact of working experience and its learning effect on the teamwork perception is shown in Table 5, revealing that before the training, staff members of difference groups of working duration showed a significant difference in teamwork perception, and those with longer working experience showed better overall score (p < 0.05). After the completion of the implementation of the program, however, the difference was eliminated that the overall perception scores became similar among the four experience groups (p = 0.56), while the changes of the perception scores by the program, i.e., the before-after differences, were not significant for all four groups of working experience.

**Table 5:** Comparison of perception ratings on teamwork concept among team members with different durations of working experience

**Years of experience**	***Before***		***After***	
	**Rating**	***p*** *	**Rating**	***p*** *
3 or less	4.97 ± 0.44	0.046	5.38 ± 1.27	0.59
4 - 10	5.02 ± 0.88		5.24 ± 0.89	
11 - 19	5.47 ± 0.63		5.35 ± 0.71	
20 or more	5.77 ± 0.52		5.67 ± 0.32	

*ANOVA.

### Outcome measurement

The teamwork skills identifies as applied in the procurement and transplantation process by the healthcare workers are summarized in Table 6. Skills documented being exhibited in the process of procurement and transplantation were found in all four categories of TRM, more in the dimension of communication and leadership, and less in mutual support.

**Table 6:** Summary of specific team behaviors related to teamwork skills documented from the case review and discussion for procurement and transplantation cases

**Dimension/Skill**	**Description of the team behavior found during reviews**
Leadership	
Briefing	The procurement leader physicians called the procurement coordinators and laboratory staff for their readiness before beginning the actual procurement process.
Huddle	The procurement leader physician confirmed the goal of action and planned the expected process with the members.
Debriefing	The procurement team discussed after completion of the procedure, stressing on unexpected conditions.
Situation Monitoring	
Situation monitoring	Staff members were trained to upload updated patient clinical data and laboratory results into the platform.
Situation awareness	Institutional patient data platform to update the progress during procurement and transplantation. This improved the situation awareness of the on-duty team members.
Shared mental model	Procurement staff, laboratory staff and procurement leaders updated for the process during organ donation.
Mutual Support	
“I’M SAFE”	Monitor the working hours to avoid working for more than 12 hours for each shift.
Communication	
Hand off (“ISBAR”)	Procurement member communicated each other with a structured form (ISBAR), which was later incorporated into the case records.
Call-out	Laboratory member called team members to confirm the information been received by the whole team.
	Critical test result (i.e., positive HIV) confirmed informed to all team members until the whole process stopped.
Time-out	Structured time-out checklist used during organ donation and transplantation, stressed on correct laboratory results.

There was no any error event or unexpected patient harm reported during the intervention period. Of note, of two potential donors during the intervention period, a positive HIV result was found, and the results were timely and correctly informed to the procurement staff by the laboratory staff, thus the donation process was terminated. No recipient was transplanted with an infected organ. No more error in the following transplantation process was noted, suggesting a marked improvement of patient safety in the organ procurement and transplantation service in this institution.

## Discussion

Out study results showed that implementation of the Team Resource Management program might be a promising method to enhance teamwork culture and patient safety in the healthcare service of organ procurement and transplantation.

After the instance of unintended HIV-positive organ transplantation event occurred in our institution [14,16], a number of measures were adopted to improve the level of patient safety, including the integration of the TRM program, as described in this report, into the multidisciplinary training and practice. In addition to the reporting of a high rate of satisfaction to the program, the participants also showed a reassuring level of perception of the TRM core concepts and understanding of the skills. The effect was seen mainly in the dimensions of teamwork framework and situation awareness, with the operation room and transplantation group demonstrating the greatest increment of perception. The findings were, at least in part, compatible with previous perspective [17,18] that surgeons who were traditionally regarded as being ranked higher in the organizational hierarchy usually also had the highest level of positive perception of teamwork, communication, and collaboration, especially in the OR settings [19,20]. Once more motivated, their active participation in the improvement program would be expected to achieve high level of success in enhance patient safety, as shown by this study.

It is notable that the organ procurement team, despite the expression of a high satisfaction rating for the TRM training program, showed a negative effect of training in term of teamwork perception. This undesired training result might partially be explained by the content of our TRM training program that during the implementation period, the central tasks were aimed at the prevention of errors related to the key process redesigning of the pertinent organ procurement procedures. As a consequence, the team members, at the centre of changing, were required to make greater modification from their original customs in terms of communication and teamwork behavior [21]. The stress was even intensified by rapid changes in working environment by the program, the undesired increase of hierarchical pressure after the incident related to procurement, and the unabated workload imposing upon them. Therefore, compatible with what reported in the literature concerning the workload or stress associated with the need to achieve effectiveness of training [22,23], the negative deviation of perception expressed by the team members for procurement might be expected and understood.

In this study we found that junior staff, although expressing relatively lower level of perception of teamwork culture before the program, tended to gain more perception by TRM training. One of the reasons to explain this might be that the senior staff had already their personal experience and would be subconsciously reluctant to change. This observation appeared in concordance with the findings in our study that the senior staff did show desired levels of understanding and acceptance of the concept of teamwork framework (category I), but also showed less willingness for changes in behavior (categories II through V). Overall, these observations were compatible with what was commonly referred as ‘the earlier the better in response’ [24,25], which was regarded as one of the basic concepts for training and education.

One of the important goals we had through this TRM program implementation was to build effective safety barriers for the organ procurement and transplantation processes by creating procedure standards and useful working sheets and checklists. We believe we have achieved this goal. After the implementation, we closely monitored the rate of compliance to these checklists, procedures and standards. In 2012, we have 67 cases of organ transplantation, who were checked in accordance with the newly revised policy and procedure, and successfully identified an anti-HIV (+) case from the potential donors, and successfully stopped the organ procurement process immediately. Furthermore, no one of near miss or violation to procedures was found. It is therefore considered that we successfully integrated TRM training program into staff training for organ procurement as well as for organ transplantation. The re-designed working environment also helped to shape a better safety climate and provided more effective safety barriers that the staff could also work together more effectively in the improvement of patient safety in organ transplantation.

There were a number of limitations in our study. First, it was a single-center experience on a specified TRM training program aiming for repairing the drawbacks [14,16] of our processes on organ procurement and transplantation. Compared with other hospital-based TRM programs on general purpose, our program was tailored for the procurement and transplantation process, and would be different that generalization of its efficacy should be considered carefully [26]. Second, the behavioral changes affected by the learning and training of skills during the program might not be limited only to the findings on the audit of documents from the meetings and discussions, although by audit we were comfortable with the fruitful findings of behavioral changes. As a complement tool, a simulation test might be also useful to further evaluate the training result. In face of the above limitations for our study, we recommend that further studies might be required to confirm the role of TRM training program in helping the healthcare system to improve the overall safety culture.

## Conclusion

In conclusion, integration of Team Resource Management program into the staff training is a promising method to improve the teamwork skills and behavior, as well as to improve patient safety in organ procurement and transplantation. Endeavor in the establishment of teamwork culture might be a keystone step to the improvement of patient safety in this complex healthcare process.

## Abbreviations

TRM: Team resource management; OR: Operating room; HIV: Human immunodeficiency virus; TORSC: Taiwan organ registry and sharing center.

## Competing interests

The authors declare that they have no competing interests.

## Authors’ contributions

HYC designed the TRM training program, collected the data and performed the data analysis. JJS participated in the design of training program and analysis of the study data, and helped to draft the manuscript. CCW organized the TRM training and designed the questionnaire. CLC participated in the video training and assessment of TRM skills. HMY analyzed the data of satisfaction questionnaire interview. HSF participated in the design of the study and organized the case review process. LYP organized the TRM course and participated in the analysis of TRM skills. HKY conceived the study and participated the drafting of the manuscript. All authors read and approved the final manuscript.

## Pre-publication history

The pre-publication history for this paper can be accessed here:

http://www.biomedcentral.com/1471-2482/14/51/prepub

## Supplementary Material

Additional file 1 Questionnaire used in this study to evaluate the patient safety culture and learning perception about TRM for all participants, originally prepared in Chinese. Click here for file

Additional file 2 English translation version of the Questionnaire. Click here for file

## References

[B1] Garrouste-Orgeas M Philippart F Bruel C Max A Lau N Misset B Overview of medical errors and adverse events Ann Intensive Care 2012 2 2 22339769 10.1186/2110-5820-2-2 PMC3310841

[B2] Hayashino Y Utsugi-Ozaki M Feldman MD Fukuhara S Hope modified the association between distress and incidence of self-perceived medical errors among practicing physicians: prospective cohort study PLoS One 2012 7 e35585 22530055 10.1371/journal.pone.0035585 PMC3329473

[B3] Pham JC Aswani MS Rosen M Lee HW Huddle M Weeks K Pronovost PJ Reducing medical errors and adverse events Ann Rev Med 2012 63 447 463 22053736 10.1146/annurev-med-061410-121352

[B4] Schriefer J Leonard MS Patient safety and quality improvement: an overview of QI Pediatr Rev 2012 33 353 359 22855927 10.1542/pir.33-8-353

[B5] Rosenstein AH Measuring and managing the economic impact of disruptive behaviors in the hospital J Healthc Risk Manag 2010 30 20 26 20979161 10.1002/jhrm.20049

[B6] Lawton R McEachan RRC Giles SJ Sirriyeh R Watt IS Wright J Development of an evidence-based framework of factors contributing to patient safety incidents in hospital settings: a systematic review BMJ Qual Saf 2012 21 369 380 10.1136/bmjqs-2011-000443 PMC3332004 22421911

[B7] Blendon RJ Desroches CM Brodie M Benson JM Rosen AB Shneider E Altman D Zapert K Merrmann MJ Steffenson AE Views of practicing physicians and the public on medical errors N Engl J Med 2002 347 1933 1940 12477944 10.1056/NEJMsa022151

[B8] Carayon P Wood KE Patient safety: the role of human factors and systems engineering Inf Knowledge Sys Manag 2009 8 23 46 PMC3057365 20543237

[B9] Agency for Healthcare Research and Quality TeamSTEPPS instructor guide 2006 Rockville, Md: AHRQ AHRQ Publication No. 06-0020-0

[B10] Scanlon MC Karsh BT Value of human factors to medication and patient safety in the intensive care unit Crit Care Med 2010 38 6 Suppl S90 S96 20502180 10.1097/CCM.0b013e3181dd8de2 PMC4455947

[B11] McKeon LM Cunningham PD Oswaks JS Improving patient safety: patient-focused, high-reliability team training J Nurs Care Qual 2009 24 76 82 19092483 10.1097/NCQ.0b013e31818f5595

[B12] Morag I Gopher D Spillinger A Auerbach-Shpak Y Laufer N Lavy Y Milwidsky A Feigin RR Pollack S Maza I Azzam ZS Admi H Soudry M Human factors-focused reporting system for improving care quality and safety in hospital wards Hum Factors 2012 54 195 213 22624287 10.1177/0018720811434767

[B13] Grogan EL Stiles RA France DJ Speroff T Morris JA Nixon B Gaffney FA Seddon R Pinson QW The impact of aviation-based teamwork training on the attitudes of health-care professionals J Am Coll Surg 2004 199 843 848 15555963 10.1016/j.jamcollsurg.2004.08.021

[B14] Ison MG Holl JL Ladner D Preventable errors in organ transplantation: an emerging patient safety issue? Am J Transplant 2012 12 2307 2312 22703471 10.1111/j.1600-6143.2012.04139.x PMC3429784

[B15] Bellandi T Albolino S Tartaglia R Filipponi F Unintended transplantation of three organs from an HIV-positive donor: report of the analysis of an adverse event in a regional health care service in Italy Transplant Proc 2010 42 2187 2189 20692439 10.1016/j.transproceed.2010.05.034

[B16] The Associated Press Taiwan–5 Get Organs with HIV The New York Times 2011 New York City: Associated Press

[B17] Sexton JB Thomas EJ Helmreich RL Error, stress, and teamwork in medicine and aviation: cross sectional surveys Brit Med J 2000 320 745 749 10720356 10.1136/bmj.320.7237.745 PMC27316

[B18] Levy SM Senter CE Hawkins RB Zhao JY Doody K Kao LS Lally KP Tsao K Implementing a surgical checklist: more than checking a box Surgery 2012 152 331 336 22770952 10.1016/j.surg.2012.05.034

[B19] Conley DM Singer SJ Edmondson L Berry WR Gawande AA Effective surgical safety checklist implementation J Am Coll Surg 2011 212 873 879 21398154 10.1016/j.jamcollsurg.2011.01.052

[B20] Thomassen O Brattebø G Heltne JK Søfteland E Espeland A Checklists in the operating room: Help or hurdle? A qualitative study on health workers’ experiences BMC Health Serv Res 2010 10 342 21171967 10.1186/1472-6963-10-342 PMC3009978

[B21] Smollan RK Sayers JG Organizational culture, organizational change and emotions: a qualitative study J Chang Manage 2009 9 435 457

[B22] van den Hombergh P Künzi B Elwyn G van Doremalen J Akkermans R Grol R Wensing M High workload and job stress are associated with lower practice performance in general practice: an observational study in 239 general practices in the Netherlands BMC Health Serv Res 2009 9 118 19604386 10.1186/1472-6963-9-118 PMC2720387

[B23] Goitein L Shanafelt TD Wipf JE Slatore CG Back AL The effects of work-hour limitations on resident well-being, patient care, and education in an internal medicine residency program Arch Intern Med 2005 165 2601 2606 16344417 10.1001/archinte.165.22.2601

[B24] Melnyk BM Fineout-Overholt E Gallagher-Ford L Kaplan L The state of evidence-based practice in US nurses: critical implications for nurse leaders and educators J Nurs Adm 2012 42 410 417 22922750 10.1097/NNA.0b013e3182664e0a

[B25] Caldwell DF Chatman J O’Reilly CA 3rd Ormiston M Lapiz M Implementing strategic change in a health care system: the importance of leadership and change readiness Health Care Manage Rev 2008 33 124 133 18360163 10.1097/01.HMR.0000304501.82061.e0

[B26] Musson DM Helmreich RL Team training and resource management in health care: current issues and future directions Harvard Health Policy Rev 2004 5 25 35

