# Supplementary material for: Integrating team resource management program into staff training improves staff’s perception and patient safety in organ procurement and transplantation: the experience in a university-affiliated medical center in Taiwan
Source: BMC Surg. 2014 Aug 11;14:51. doi: 10.1186/1471-2482-14-51 (PMC4136399; doi:10.1186/1471-2482-14-51)
Supplement: Additional file 1 — Questionnaire used in this study to evaluate the patient safety culture and learning perception about TRM for all participants, originally prepared in Chinese. [file 1471-2482-14-51-S1.pdf]

這是一個不記名的問卷調查，您的參與直接影響了此項調查的成功與否，請依照您在職場上對於團隊合作的實際感受回答下列問題，問題的答案沒有所謂的對或錯，通常您的第一直覺答案會是最好的一個。您的資料將絕對的保密。

依照您對下列情形的同意程度評分，滿分為7分，1 為最低分、2 為次低分、.....、7 為最高分

評分範例：若您評分的分數為4分，則請圈選尺度4

1 2 3 4 5 6 7 (4 circled)

非常不同意 → 非常同意

範例題目：	對團隊合作的實際感受
我們會將犯錯也視為是一種讓我們成長與學習的機會。	1 2 3 4 5 6 7 (7 circled)<br>非常不同意 → 非常同意

解說：我非常同意目前職場上的團隊合作有做到此目標，則分數為7分。

第一部份：醫療團隊資源管理訓練之需求評估

構面一

	對團隊合作的實際感受
1. 詢問病人及其家屬對於醫療照護的意見是重要的事。	1 2 3 4 5 6 7
2. 把病人及其家屬當作醫療照護團隊的重要一員。	1 2 3 4 5 6 7
3. 醫院的行政支持有助於照護團隊的成功。	1 2 3 4 5 6 7
4. 團隊的目標比個人的目標更為重要。	1 2 3 4 5 6 7
5. 優秀的團隊成員較能想到其他成員的需要。	1 2 3 4 5 6 7
6. 優秀的醫療團隊與其他行業的優秀團隊具有類似的特質。	1 2 3 4 5 6 7
7. 團隊成員都能獲得適當的訓練使團隊能有效地運作。	1 2 3 4 5 6 7
8. 團隊有足夠的人力和能力來達成目標。	1 2 3 4 5 6 7
9. 團隊中沒有任何個人、小團體或是單一性別能左右團隊的行動。	1 2 3 4 5 6 7
10. 團隊具有正面的自我形象。	1 2 3 4 5 6 7

評分範例：若您評分的分數為4分，則請圈選尺度4。	1 2 3 (4) 5 6 7<br>非常不同意 ——→ 非常同意

構面二

	對團隊合作的實際感受
1. 團隊的領導者很重視團隊合作。	1 2 3 4 5 6 7
2. 團隊的領導者能與成員分享訊息。	1 2 3 4 5 6 7
3. 團隊的領導者會利用各種不同的機會與成員分享訊息。	1 2 3 4 5 6 7
4. 團隊的領導者認為勇於認錯是一種學習契機。	1 2 3 4 5 6 7
5. 團隊的領導者的責任之一是作為所有成員行為的典範。	1 2 3 4 5 6 7
6. 團隊的成員能主動了解自己的角色及職責。	1 2 3 4 5 6 7
7. 團隊的成員能主動了解其他成員的角色及職責。	1 2 3 4 5 6 7
8. 團隊的成員都能清楚了解每個成員正在進行的工作並能避免工作重複。	1 2 3 4 5 6 7
9. 當團隊成員的角色及職責改變時，會有特定的計畫或訓練協助其勝任新的角色與職責。	1 2 3 4 5 6 7
10. 雖然已經有一正式的團隊領導者，但領導功能會視情況、任務的需求和團隊成員的技能而適時地調整。	1 2 3 4 5 6 7
11. 團隊的領導者會教導及支持團隊成員。	1 2 3 4 5 6 7
12. 團隊成員的意見會被充分重視。	1 2 3 4 5 6 7

構面三

	對團隊合作的實際感受
1. 工作中如果有不明瞭的地方，很容易可以找到人詢問。	1 2 3 4 5 6 7
2. 有關診斷及治療所需的關鍵資訊，可以適時提供給我。	1 2 3 4 5 6 7
3. 團隊成員會被教導如何有系統地察覺環境中的重要變化。	1 2 3 4 5 6 7

評分範例：若您評分的分數為4分，則請圈選尺度4。	1 2 3 (4) 5 6 7<br>非常不同意 → 非常同意

	對團隊合作的實際感受
4. 照護病人時，可以充分得到所需要的支援。	1 2 3 4 5 6 7
5. 每位成員對於團隊的貢獻都會被重視。	1 2 3 4 5 6 7
6. 為了有效地完成工作，團隊成員間會互相了解彼此的工作性質。	1 2 3 4 5 6 7
7. 團隊成員間會使用有效的決策過程以達成共識並解決問題。	1 2 3 4 5 6 7
8. 團隊成員能彼此互相信任。	1 2 3 4 5 6 7
9. 團隊的其他成員能給我建設性的回饋。	1 2 3 4 5 6 7
10. 當臨床上的意見出現分歧時，團隊成員間會以病人最大利益為前提來解決問題。	1 2 3 4 5 6 7
11. 當發生紛爭時，團隊成員會迅速地解決紛爭。	1 2 3 4 5 6 7

構面四

	對團隊合作的實際感受
1. 監測病人狀況有助於提昇團隊的表現與成效。	1 2 3 4 5 6 7
2. 注意其他團隊成員的情緒及身體狀況是工作中重要的一環。	1 2 3 4 5 6 7
3. 主動幫忙過於疲勞或壓力太大而無法執行任務的團隊成員是理所當然。	1 2 3 4 5 6 7
4. 注意自己的的情緒和身體狀態能使工作更有效率。	1 2 3 4 5 6 7
5. 團隊成員間互相支援是一個提高團隊績效的有效方法。	1 2 3 4 5 6 7
6. 如果團隊成員間無法有效的溝通，則會增加犯錯的風險。	1 2 3 4 5 6 7
7. 溝通不良是醫療錯誤中常見的原因。	1 2 3 4 5 6 7
8. 建立與病人和家屬溝通聯繫的管道可以減少醫療不良事件的發生。	1 2 3 4 5 6 7
9. 我樂於和會提問的團隊成員一起工作。	1 2 3 4 5 6 7

評分範例：若您評分的分數為4分，則請圈選尺度4。	1 2 3 ④ 5 6 7<br>非常不同意 → 非常同意

構面五

	對團隊合作的實際感受
1. 稱職的臨床工作者從不會犯錯。	1 2 3 4 5 6 7
2. 資淺的團隊成員不會質疑資深團隊成員所做的決定。	1 2 3 4 5 6 7
3. 護士不會質疑醫師所做的決定。	1 2 3 4 5 6 7
4. 疲勞不影響我工作的效率。	1 2 3 4 5 6 7
5. 即便在工作中被干擾，也不會影響到我所照顧的病人的安全。	1 2 3 4 5 6 7
6. 即使當我感到壓力和分心時，也不影響我的工作效率。	1 2 3 4 5 6 7

情緒管理

下列各項問題是關於您目前在職場上的感覺及感受，請針對每一問題選一最接近您感覺的答案。在職場中有多少時候會...

	總是如此	經常如此	偶爾如此	甚少如此	從不如此
1. 我容易感到壓力。	☐	☐	☐	☐	☐
2. 我大部分的時候是輕鬆自在的。	☐	☐	☐	☐	☐
3. 我常常會擔心很多事情。	☐	☐	☐	☐	☐
4. 我很少覺得憂鬱。	☐	☐	☐	☐	☐
5. 我很容易感到困惑。	☐	☐	☐	☐	☐
6. 我很容易覺得失望。	☐	☐	☐	☐	☐
7. 我經常改變心情。	☐	☐	☐	☐	☐
8. 我經常會有情緒的波動。	☐	☐	☐	☐	☐
9. 我容易被激怒。	☐	☐	☐	☐	☐
10. 我常覺得很憂鬱。	☐	☐	☐	☐	☐

第二部分：個人基本資料

1. 性別：□1.男　□2.女

2. 出生年次：民國 ＿＿＿ 年

3. 您的宗教信仰：
□1.無　□2.佛教　□3.道教　□4.回教　□5.天主教　□6.基督教
□7.一貫道　□8.其他＿＿＿＿

4. 服務醫院：
□1.醫學中心　□2.區域教學醫院　□3.區域醫院
□4.地區教學醫院　□5.地區醫院　□6.基層診所

5. 醫院所在地：
□1.北部(宜蘭/基隆/台北/桃園/新竹)　□2.中部(苗栗/台中/南投/彰化)
□3.南部(雲林/嘉義/台南/高雄/屏東)　□4.東部(花蓮/台東)
□5.離島(澎湖/金門/馬祖)

6. 工作性質：
□1.主治醫師　□2.住院醫師　□3.護理師　□4.專科護理師
□5.護士　□6.其他醫療人員 ＿＿＿＿＿＿

7. 是否為主管：□1.否　□2.是

8. 服務科別：
□1.內科　□2.外科　□3.婦產科　□4.小兒科　□5.骨科
□6.眼科　□7.耳鼻喉科　□8.皮膚科　□9.復健科　□10.泌尿科
□11.精神科　□12.神經科　□13.急診醫學科　□14.牙科　□15.麻醉科
□16.家庭醫學科　□17.腫瘤科　□18.其他 ＿＿＿＿＿＿

9. 服務年資：＿＿＿年＿＿＿月（自開始進入臨床工作/執業起）

10. 進入現職機構之年資：＿＿＿年＿＿＿月

11. 主要工作地點：
□1.加護病房　□2.急診　□3.一般病房　□4.門診　□5.手術室/開刀房
□6.其他 ＿＿＿＿＿

12. 在過去一年內，您是否接受過相關議題之在職教育訓練：
□1.否
□2.是：＿＿＿次，題目為＿＿＿＿＿＿＿＿＿＿＿＿＿＿＿＿＿＿＿＿

13. 依您的經驗中，是否擔任過團隊領導者？
□1.從未有過　□2.很少　□3.偶爾　□4.經常　□5.總是

問卷業已填畢，敬請回傳予聯絡人

再次感謝您的配合調查!
敬祝您健康快樂!
