# Supplementary material for: Integrating team resource management program into staff training improves staff’s perception and patient safety in organ procurement and transplantation: the experience in a university-affiliated medical center in Taiwan
Source: BMC Surg. 2014 Aug 11;14:51. doi: 10.1186/1471-2482-14-51 (PMC4136399; doi:10.1186/1471-2482-14-51)
Supplement: Additional file 2 — English translation version of the Questionnaire. [file 1471-2482-14-51-S2.pdf]

Part I: Assessment of the medical team resource management training

Domain I: Teamwork framework

	Level of Agreement
1. It is important to ask the patients and their families for opinions about the care they receive.	○ 1 ○ 2 ○ 3 ○ 4 ○ 5 ○ 6 ○ 7
2. The patients and their families are as important as the medical care team.	○ 1 ○ 2 ○ 3 ○ 4 ○ 5 ○ 6 ○ 7
3. Support for the hospital is beneficial for the team's success.	○ 1 ○ 2 ○ 3 ○ 4 ○ 5 ○ 6 ○ 7
4. The goal of the team is more important than the individual goals.	○ 1 ○ 2 ○ 3 ○ 4 ○ 5 ○ 6 ○ 7
5. Excellent team members more often think of the need from other members of the team.	○ 1 ○ 2 ○ 3 ○ 4 ○ 5 ○ 6 ○ 7
6. Excellent medical team shares similar characteristics with outstanding teams of other fields.	○ 1 ○ 2 ○ 3 ○ 4 ○ 5 ○ 6 ○ 7
7. Team members receive appropriate training so that the team can operate effectively.	○ 1 ○ 2 ○ 3 ○ 4 ○ 5 ○ 6 ○ 7
8. The team has enough manpower and ability to achieve goals.	○ 1 ○ 2 ○ 3 ○ 4 ○ 5 ○ 6 ○ 7
9. The action of the team is not affected by the decision from a single team member or small group within it.	○ 1 ○ 2 ○ 3 ○ 4 ○ 5 ○ 6 ○ 7
10. There is a positive self-image of the team.	○ 1 ○ 2 ○ 3 ○ 4 ○ 5 ○ 6 ○ 7

Domain II. Leadership

	Level of Agreement
1. The team leader emphasizes much about teamwork practice.	○ 1 ○ 2 ○ 3 ○ 4 ○ 5 ○ 6 ○ 7
2. The team leader is willing to share information with the members.	○ 1 ○ 2 ○ 3 ○ 4 ○ 5 ○ 6 ○ 7
3. Team leader uses a variety of opportunities to share information with members	○ 1 ○ 2 ○ 3 ○ 4 ○ 5 ○ 6 ○ 7
4. The team leader believes that admitting errors is an opportunity for learning.	○ 1 ○ 2 ○ 3 ○ 4 ○ 5 ○ 6 ○ 7
5. One of the responsibilities of a team leader is the acting as a role model for the members.	○ 1 ○ 2 ○ 3 ○ 4 ○ 5 ○ 6 ○ 7
6. Members of the team take the initiative to understand their own roles and responsibilities in the team.	○ 1 ○ 2 ○ 3 ○ 4 ○ 5 ○ 6 ○ 7
7. Members of the team take the initiative to understand the roles and responsibilities of the other members of the team.	○ 1 ○ 2 ○ 3 ○ 4 ○ 5 ○ 6 ○ 7
8. Every team member has a clear understanding of the ongoing work of each member and avoids overlapping of the tasks.	○ 1 ○ 2 ○ 3 ○ 4 ○ 5 ○ 6 ○ 7
9. When there is a change of the role or duty of any team member, there is a specific program or training to	○ 1 ○ 2 ○ 3 ○ 4 ○ 5 ○ 6 ○ 7

enhance the competence.	
10. Although there is a formal leader, but the actual function of the leader is adjusted according to the situation, mission and the skills of the team members.	◌ 1 ◌ 2 ◌ 3 ◌ 4 ◌ 5 ◌ 6 ◌ 7
11. The team leader teaches and supports the team members	◌ 1 ◌ 2 ◌ 3 ◌ 4 ◌ 5 ◌ 6 ◌ 7
12. Opinions from the team members are regarded as important information.	◌ 1 ◌ 2 ◌ 3 ◌ 4 ◌ 5 ◌ 6 ◌ 7

Domain III. Situation awareness

	Level of Agreement
1. I can easily obtain information from others from the team when I encounter unclear condition.	◌ 1 ◌ 2 ◌ 3 ◌ 4 ◌ 5 ◌ 6 ◌ 7
2. The key information regarding diagnosis and treatment is timely available.	◌ 1 ◌ 2 ◌ 3 ◌ 4 ◌ 5 ◌ 6 ◌ 7
3. Team members will be taught how to systematically detect important changes in the environment.	◌ 1 ◌ 2 ◌ 3 ◌ 4 ◌ 5 ◌ 6 ◌ 7
4. I receive full support when I am caring the patients.	◌ 1 ◌ 2 ◌ 3 ◌ 4 ◌ 5 ◌ 6 ◌ 7
5. The contribution of each member of the team will be taken seriously.	◌ 1 ◌ 2 ◌ 3 ◌ 4 ◌ 5 ◌ 6 ◌ 7
6. Team members understand each other's task to effectively complete the work.	◌ 1 ◌ 2 ◌ 3 ◌ 4 ◌ 5 ◌ 6 ◌ 7
7. Team members use effective decision-making process to reach a consensus and resolve problems.	◌ 1 ◌ 2 ◌ 3 ◌ 4 ◌ 5 ◌ 6 ◌ 7
8. There is mutual trust among the team members.	◌ 1 ◌ 2 ◌ 3 ◌ 4 ◌ 5 ◌ 6 ◌ 7
9. Other members of the team give me constructive feedbacks.	◌ 1 ◌ 2 ◌ 3 ◌ 4 ◌ 5 ◌ 6 ◌ 7
10. The team members solve the problem based on the best interests for the patients when there are conflicting opinions among the members.	◌ 1 ◌ 2 ◌ 3 ◌ 4 ◌ 5 ◌ 6 ◌ 7
11. The team members act rapidly to solve the problem when conflicting opinions are present.	◌ 1 ◌ 2 ◌ 3 ◌ 4 ◌ 5 ◌ 6 ◌ 7

Domain IV. Communication

	Level of Agreement
1. Monitoring of the patient's condition is beneficial for improving the team's performance and effectiveness.	◌ 1 ◌ 2 ◌ 3 ◌ 4 ◌ 5 ◌ 6 ◌ 7
2. It is important during work to note the emotional and physical conditions of other members of the team.	◌ 1 ◌ 2 ◌ 3 ◌ 4 ◌ 5 ◌ 6 ◌ 7
3. It is taken for granted to actively help other members who suffer from tiredness or too much pressure to perform the task.	◌ 1 ◌ 2 ◌ 3 ◌ 4 ◌ 5 ◌ 6 ◌ 7
4. I constantly pay attention to my level of emotional and physical to work more efficiently.	◌ 1 ◌ 2 ◌ 3 ◌ 4 ◌ 5 ◌ 6 ◌ 7
5. It is an effective method to improve team performance by mutually supporting other members of the team.	◌ 1 ◌ 2 ◌ 3 ◌ 4 ◌ 5 ◌ 6 ◌ 7

6. The risk of making errors increases if there is lack of effective communication among the members.	◌ 1 ◌ 2 ◌ 3 ◌ 4 ◌ 5 ◌ 6 ◌ 7
7. Poor communication is a common cause of medical errors.	◌ 1 ◌ 2 ◌ 3 ◌ 4 ◌ 5 ◌ 6 ◌ 7
8. The occurrence of adverse event can be avoided by build the communication channels with the patient and family.	◌ 1 ◌ 2 ◌ 3 ◌ 4 ◌ 5 ◌ 6 ◌ 7
9. I am happy to work together with other members of the team who actively raise questions during work.	◌ 1 ◌ 2 ◌ 3 ◌ 4 ◌ 5 ◌ 6 ◌ 7

Domain V. Mutual support

	Level of Agreement
1. Competent healthcare workers never make mistakes.	◌ 1 ◌ 2 ◌ 3 ◌ 4 ◌ 5 ◌ 6 ◌ 7
2. Junior team members do not question the decisions from the senior members.	◌ 1 ◌ 2 ◌ 3 ◌ 4 ◌ 5 ◌ 6 ◌ 7
3. Nurses do not question the decisions from the physicians.	◌ 1 ◌ 2 ◌ 3 ◌ 4 ◌ 5 ◌ 6 ◌ 7
4. My performance during work is not affected by fatigue.	◌ 1 ◌ 2 ◌ 3 ◌ 4 ◌ 5 ◌ 6 ◌ 7
5. The safety of my patients is not affected by the interference when I am working.	◌ 1 ◌ 2 ◌ 3 ◌ 4 ◌ 5 ◌ 6 ◌ 7
6. My performance during work is not affected when I feel pressure and distraction.	◌ 1 ◌ 2 ◌ 3 ◌ 4 ◌ 5 ◌ 6 ◌ 7

Emotional Management

	Level of Agreement
1. I frequently fell pressure.	◌ 1 ◌ 2 ◌ 3 ◌ 4 ◌ 5 ◌ 6 ◌ 7
2. I feel easy mot of the time.	◌ 1 ◌ 2 ◌ 3 ◌ 4 ◌ 5 ◌ 6 ◌ 7
3. I am frequently worried about something.	◌ 1 ◌ 2 ◌ 3 ◌ 4 ◌ 5 ◌ 6 ◌ 7
4. I seldom feel not well.	◌ 1 ◌ 2 ◌ 3 ◌ 4 ◌ 5 ◌ 6 ◌ 7
5. I am frequently confused.	◌ 1 ◌ 2 ◌ 3 ◌ 4 ◌ 5 ◌ 6 ◌ 7
6. I frequently got disappointed.	◌ 1 ◌ 2 ◌ 3 ◌ 4 ◌ 5 ◌ 6 ◌ 7
7. I mood frequently changes.	◌ 1 ◌ 2 ◌ 3 ◌ 4 ◌ 5 ◌ 6 ◌ 7
8. I have frequent fluctuation of the emotion.	◌ 1 ◌ 2 ◌ 3 ◌ 4 ◌ 5 ◌ 6 ◌ 7
9. I am offended frequently.	◌ 1 ◌ 2 ◌ 3 ◌ 4 ◌ 5 ◌ 6 ◌ 7
10. I have depression frequently.	◌ 1 ◌ 2 ◌ 3 ◌ 4 ◌ 5 ◌ 6 ◌ 7

Part II：Baseline Data during Work (Abridged)

1. Sex
2. Age
3. Experience as a manager: in year
4. Experience of healthcare service: in year
5. Location of healthcare work
6. Experience as a team leader: never; seldom; occasional; often; always
